# The relation between *Blastocystis* and the intestinal microbiota in Swedish travellers

**DOI:** 10.1186/s12866-017-1139-7

**Published:** 2017-12-11

**Authors:** Joakim Forsell, Johan Bengtsson-Palme, Martin Angelin, Anders Johansson, Birgitta Evengård, Margareta Granlund

**Affiliations:** 1 Department of Clinical Microbiology, Umeå University, -901 87 Umeå, SE Sweden; 2 Department of Infectious Diseases, Institute of Biomedicine, The Sahlgrenska Academy, University of Gothenburg, Gothenburg, Sweden; 3 Centre for Antibiotic Resistance Research (CARe), University of Gothenburg, Gothenburg, Sweden; 4 Department of Clinical Microbiology, Infectious Diseases, Umeå University, Umeå, Sweden; 5 Department of Clinical Microbiology, Bacteriology, and the Laboratory for Molecular Infection Medicine Sweden, Umeå University, Umeå, Sweden

**Keywords:** *Blastocystis*, Subtype, Persistence, Travel, Microbiota, *Sporolactobacillus*, *Candidatus* Carsonella, Transmission

## Abstract

**Background:**

*Blastocystis* sp. is a unicellular eukaryote that is commonly found in the human intestine. Its ability to cause disease is debated and a subject for ongoing research. In this study, faecal samples from 35 Swedish university students were examined through shotgun metagenomics before and after travel to the Indian peninsula or Central Africa. We aimed at assessing the impact of travel on *Blastocystis* carriage and seek associations between *Blastocystis* and the bacterial microbiota.

**Results:**

We found a prevalence of *Blastocystis* of 16/35 (46%) before travel and 15/35 (43%) after travel. The two most commonly *Blastocystis* subtypes (STs) found were ST3 and ST4, accounting for 20 of the 31 samples positive for *Blastocystis*. No mixed subtype carriage was detected. All ten individuals with a typable ST before and after travel maintained their initial ST. The composition of the gut bacterial community was not significantly different between *Blastocystis*-carriers and non-carriers. Interestingly, the presence of *Blastocystis* was accompanied with higher abundances of the bacterial genera *Sporolactobacillus* and *Candidatus* Carsonella. *Blastocystis* carriage was positively associated with high bacterial genus richness, and negatively correlated to the *Bacteroides*-driven enterotype. These associations were both largely dependent on ST4 – a subtype commonly described from Europe – while the globally prevalent ST3 did not show such significant relationships.

**Conclusions:**

The high rate of *Blastocystis* subtype persistence found during travel indicates that long-term carriage of *Blastocystis* is common. The associations between *Blastocystis* and the bacterial microbiota found in this study could imply a link between *Blastocystis* and a healthy microbiota as well as with diets high in vegetables. Whether the associations between *Blastocystis* and the microbiota are resulting from the presence of *Blastocystis*, or are a prerequisite for colonization with *Blastocystis*, are interesting questions for further studies.

**Electronic supplementary material:**

The online version of this article (10.1186/s12866-017-1139-7) contains supplementary material, which is available to authorized users.

## Background

*Blastocystis* sp. is an anaerobic unicellular eukaryote that can reside in the gastrointestinal tract of humans and a wide variety of animals [1]. Taxonomically it belongs to the phylum Stramenopiles [2] and it is the only member of this phylum found in the human intestine. Human carriage of *Blastocystis* is common worldwide, and recent studies using molecular tools report detection rates of 22–56% in European countries [3–5] and 37–100% in Asian and African countries [6–8]. There is a high degree of genetic diversity within *Blastocystis* sp., and 17 genetic subtypes, named ST1–17, are recognized. ST1–9 and ST12 have so far been found in humans of which ST3 is the most commonly detected subtype [9, 10]. The most notable geographic difference in subtype distribution is that while ST4 is the second most common subtype in Europe it is rarely found in South America, Africa and Asia [7, 9, 10]. The effect of international travel on subtype carriage is not known. *Blastocystis* has been associated with diarrhoea, abdominal pain, and vomiting, but its role as a causative agent in human diseases is unclear and debated [11]. Research on subtype-dependent pathogenicity is ongoing but there is to date no widely accepted distinction between pathogenic and non-pathogenic subtypes [11, 12]. When *Blastocystis* is detected by microscopy or PCR in diagnostic examinations of faecal samples from humans with suspected disease, it is often not possible to determine if the finding represents acute infection or intestinal colonization. This prompts further research to better understand and characterize the nature of human *Blastocystis* carriership. In prolonged cases of diarrhoea or other gastrointestinal complaints after travel, findings of *Blastocystis* as the sole possible pathogen may be interpreted as infection and treated with metronidazole, trimethroprim- sulfamethoxazole and/or other antimicrobial drugs [11]. Treatment failure is common and eradication of *Blastocystis* can be difficult to achieve [13]. It is unknown whether this is due to poor drug efficacy, a failure of the host defence in eliminating a pathogen despite an appropriate drug effect, or if persistent *Blastocystis* findings in symptomatic disease signify that this organism constitutes a normal and stable part of the gut microbiota. To be able to determine the factors that govern the presence of *Blastocystis* in the human intestine, an essential step is to examine the relationships between *Blastocystis* and the bacterial gut microbiota. The aim of this study was to use metagenomics to investigate the prevalence and temporal stability of *Blastocystis* subtypes and its relation to the bacterial intestinal microbiota of Swedish university students travelling to the Indian peninsula or Central Africa.

## Methods

### Data collection

Participants in this study were part of a larger study in which Swedish students from the Universities in Umeå, Stockholm and Gothenburg travelling for international exchange studies submitted faecal samples for laboratory analyses before and after travel. The material has previously been used in a study on the acquisition of extended spectrum beta-lactamase (ESBL) producing bacteria through travel [14]. The present metagenomic study was planned and executed together with authors of a study in which faecal samples were examined for antibiotic resistance genes [15]. Included in these metagenomic studies were 35 health care students, 26/35 female, age 23–34 years, who travelled to Central Africa (17/35) or the Indian peninsula (18/35) with a median travel duration of 34 days (range 14 to 150). The students included reported no antibiotic treatment during travel or within six months prior to sampling. Faecal samples were frozen on arrival to the laboratory and DNA was extracted from frozen faeces using the QIAamp DNA stool mini kit (Qiagen). Metagenomic sequencing was performed on a HiSeq 2000 (Illumina). The sequence data, consisting of 40 to 178 million filtered read pairs per sample, was deposited in the European Nucleotide Archive as project PRJEB7369 [15].

### Bioinformatic analysis

Quality filtering of the reads and removal of host sequences was performed as previously described [15]. The read pairs of the sequence data were searched for small subunit ribosomal DNA (SSU-rDNA) sequences using Metaxa2 version 2.0 [16] (additional option “--align none”). The genus rDNA counts for each library were normalized to the total number of rDNA counts in that library, yielding relative abundances for each genus. Partial *Blastocystis* SSU-rDNA sequences and their associated subtype classifications were downloaded from the Blastocystis Sequence Typing website (http://pubmlst.org/blastocystis/) on 2014–10-15. SSU-rDNA sequences in the gut metagenomes classified as deriving from the *Blastocystis* genus by Metaxa2 were extracted and used as queries for BLAST searches [17] against the Blastocystis Sequence Typing database (options “-p blastn -e 1e-5 -m 8 -F F”), and thereby classified to subtypes. Sequence reads that could have originated from more than one subtype were noted as non-discriminatory matches and were not used in the majority of subsequent analysis. To avoid noise, subtypes with less than three reads assigned to them were removed from each library. The detection of the *Blastocystis* SSU-rDNA by the Metaxa2 tool was confirmed by testing with real-time PCR (qPCR) for *Blastocystis* [7] in which 30 of 31 samples positive according to the metagenomic analysis were positive also by qPCR.

### Detection of intestinal parasites

All samples were tested for the presence of intestinal parasites by light microscopy after formol-ether-concentration and by qPCR for the detection of *Giardia intestinalis*, *Cryptosporidium* spp., *Entamoeba histolytica* and *Entamoeba dispar* [7]. Detection of bacterial and viral enteropathogens was not performed.

### Statistical analysis

Alpha diversity (richness, Shannon and Simpson indices) was assessed using the R package Vegan (http://cran.r-project.org/web/packages/vegan/). Vegan was also used for rarefaction analysis. To determine if there was a connection between human gut bacterial composition and the *Blastocystis* subtypes present in the gut, we performed a principal component analysis (PCA) of the relative abundances of bacterial genera, as well as a principal coordinates analysis (PCoA) of the Bray-Curtis dissimilarity matrix, in the software R [18]. Prior to PCA a variance stabilizing transform (square-root transformation) of the relative abundances of bacterial genera was applied, and then the R command prcomp was used for the PCA. Spearman correlation was used to identify relationships between the relative abundances of individual bacterial genera and the total *Blastocystis* abundance or the abundance of the respective subtypes. *P*-values for correlations were adjusted for multiple testing using the Benjamini-Krieger-Yekuteli modification of the false discovery rate (FDR) [19] and tests with an FDR < 0.05 were considered statistically significant. These correlations were calculated both before and after travel, as well as on all samples together, and only correlations that were significant in all three cases have been reported, as those seem to be stable both between individuals and over time. The association between *Blastocystis* presence and bacterial diversity was assessed by comparing the mean bacterial genus richness of *Blastocystis* positive and negative samples using a two-tailed Student’s *t*-test assuming unequal variance. Relationships between *Blastocystis* positivity/negativity and the relative abundance of the *Bacteroides, Sporolactobacillus* and *Candidatus* Carsonella genera were tested in the same manner. Those tests were also carried out separately for individuals positive or negative for subtypes ST3 and ST4. Furthermore, the effect of *Blastocystis* and/or specific subtype presence or absence on total community composition was assessed on the family and phylum levels using the metaxa2_uc tool, part of the Metaxa2 Diversity Tools [20] (options “-g auto --table T --matrix T” and “-g auto --table T --matrix T -c groups”). This tool iteratively calculates Bray-Curtis dissimilarity between subsamples of communities to assess whether they are significantly dissimilar (i.e. if the distribution of taxa in a sample could have been drawn from the collective pool of taxa of a group of samples).

## Results

**Fig. 1:**
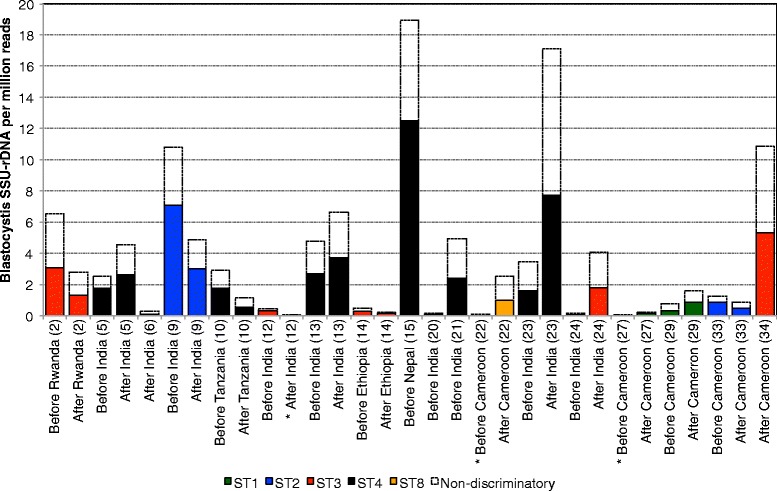
Abundance of *Blastocystis* SSU rDNA in positive individuals, before and after travel. Labels show the time point of sampling, destination, and participants’ number of inclusion. Asterisks indicate samples in which no subtype specific reads were detected

Sixteen out of the 35 subjects (46%) were positive for *Blastocystis* before travel and 15 (43%) were positive after travel based on the detection of *Blastocystis* SSU-rDNA in the metagenomic data. Eighteen (51%) were positive for *Blastocystis* either before or after travel and 13 (37%) were positive both before and after travel (Fig. 1). The relative abundance of *Blastocystis* SSU-rDNA reads varied substantially between individuals, and also between time points, ranging from 0.08 to 18.8 per million reads. Between 25% and 71% of the *Blastocystis* SSU-rDNA reads in each sample matched a specific subtype. Other *Blastocystis* SSU-rDNA reads were not specific to any single subtype because of inter-subtype sequence identity in some parts of the SSU-rDNA, and were considered as non-discriminatory with regard to subtyping. After filtering out non-discriminatory reads we did not find any evidence of co-occurrence of several different subtypes within the same individual. Instead, all SSU-rDNA reads that could be assigned to a subtype corresponded to a single variant in each individual. Subtyping was not successful in three samples in which only a few reads of non-discriminatory sequences were detected. In total, we found ST1 in 3 samples from 2 individuals, ST2 in 4 samples from 2 individuals, ST3 in 10 samples from 7 individuals, ST4 in 10 samples from 6 individuals, and ST8 in a single sample (Fig. 1; Additional file 1: Figure S1). For the ten participants that had their *Blastocystis* subtyped both before and after travel, the same subtype was detected in both samples. The median travel duration for these individuals was 41 days (range 22 to 150). Among all participants, travellers’ diarrhoea was reported by 69% (24/35), for a median of four days [15], and among those positive for *Blastocystis* both before and after travel the corresponding figure was 77% (10/13). Parasitological analysis by light microscopy revealed no pathogenic intestinal parasites, and all samples were negative in the qPCR for *Giardia intestinalis* (using a cycle treshold value cut-off of 36), *Cryptosporidium* spp., *Entamoeba histolytica* and *Entamoeba dispar*.

**Fig. 2:**
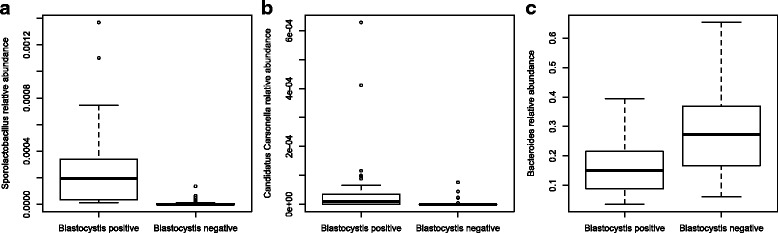
Box-plot for the relative abundance of the bacterial genera *Sporolactobacillus* (**a**), *Candidatus* Carsonella (**b**), and *Bacteroides* (**c**) in *Blastocystis* positive and negative individuals. The differences were assessed statistically through Students *t*-test

**Fig. 3:**
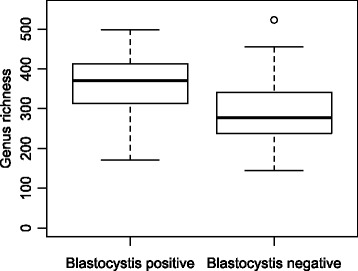
Box-plot for the bacterial genus richness in *Blastocystis* positive and negative individuals. The differences were assessed statistically through Students *t*-test

The metagenomic analysis revealed high interindividual variability of the bacterial community, but the microbiota was only moderately affected by travel (Additional file 2: Figure S2). This was previously presented by Bengtsson-Palme et al. [15] (see the supplemental material), which shows the taxonomic composition at the phylum level before and after travel to Central Africa and the Indian peninsula, respectively. In the present study, the relative abundance of *Blastocystis* was significantly positively correlated to the relative abundance of bacteria in the genera *Sporolactobacillus* and *Candidatus* Carsonella (Table 1). In addition, we found significant positive correlations between certain bacterial genera and the relative abundance of certain *Blastocystis* subtypes both before and after travel. Considering the relatively few individuals carrying each subtype we have chosen to present these findings in Additional file 3: Table S1, but have refrained from further interpretation of their importance due to small sample sizes at the subtype level. When comparing *Blastocystis* positive samples with negative samples we found positive samples to be associated with an increased relative abundance of *Sporolactobacillus* (*p* = 0.00021; Fig. 2a). An increased relative abundance of *Candidatus* Carsonella in *Blastocystis*-carriers was also noted in the total material (*p* = 0.03795; Fig. 2b), however, when samples taken before and after travel were analysed separately, this relationship was not significant (*p* = 0.1855, and *p* = 0.097, respectively). The relative abundance of *Bacteroides* was significantly lower in faecal samples containing *Blastocystis* (*p* = 0.00027; Fig. 2c). For the two most commonly found subtypes, ST3 and ST4, the negative association with *Bacteroides* was significant for ST4 (*p* = 0.005655) but not for ST3 (*p* = 0.7293). Presence of *Blastocystis* was also associated with significantly higher richness of bacterial genera (*p* = 0.0022; Fig. 3; Additional file 4: Figure S3), with this effect also being largely driven by ST4, which was significantly associated with high richness of bacterial genera (*p* = 3.65E-05), in contrast to what was found for ST3 (*p* = 0.7093).

**Table 1:** Bacterial genera with significant correlations to the abundance of overall *Blastocystis* SSU-rDNA

	Before travel		After travel		In total	
Bacteria correlated to *Blastocystis* sp. (*n* = 31)	Correlation coefficient	Adjusted *p*-value	Correlation coefficient	Adjusted *p*-value	Correlation coefficient	Adjusted *p*-value
Firmicutes; Bacilli; Bacillales; Sporolactobacillaceae; Sporolactobacillus	0.9062	< 0.0001	0.8448	< 0.0001	0.8731	< 0.0001
Proteobacteria; Gammaproteobacteria; Enterobacteriales; Enterobacteriaceae; Candidatus Carsonella	0.6148	0.0811	0.6617	0.0172	0.6430	< 0.0001

**Fig. 4:**
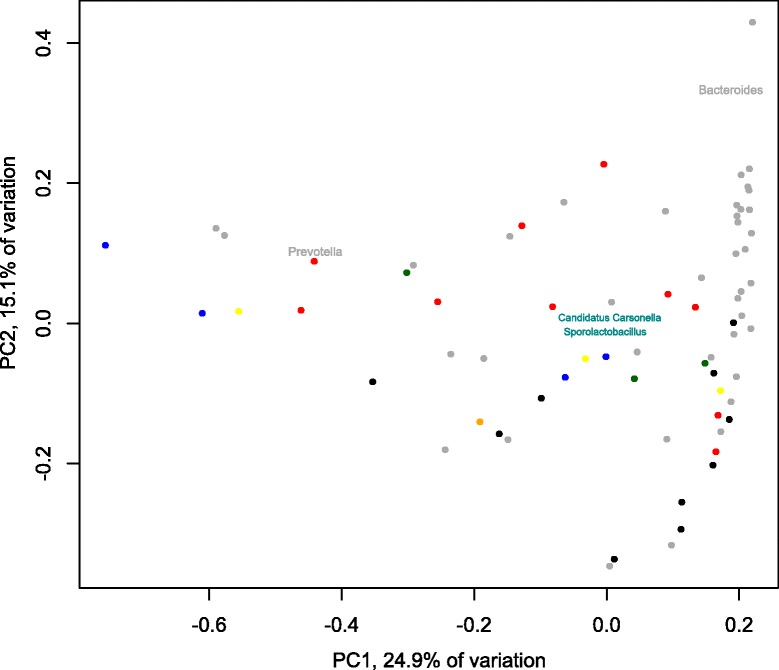
Principal component analysis of bacterial genus composition (square-root transformed abundances). Samples are colour coded by the subtype present (ST1: green; ST2: blue; ST3: red; ST4: black; ST8: orange; Ambiguous: yellow; No *Blastocystis* detected: grey). The genera *Bacteroides* and *Prevotella*, indicative of the human enterotypes, are shown as drivers of the PCA separation. In addition, the two genera significantly correlated to *Blastocystis* abundance (*Candidatus* Carsonella and *Sporolactobacillus*) are indicated in light blue

**Fig. 5:**
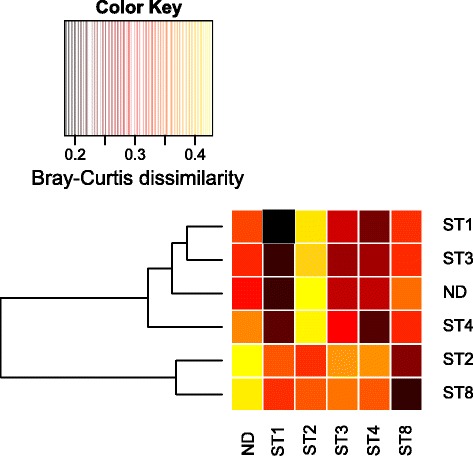
Similarity between the bacterial community compositions of individuals carrying different *Blastocystis* subtypes, or no *Blastocystis* at all (ND). Rows indicate the dissimilarity of the average community of each sample group to the average community of the sample groups of the columns, as assessed by repeated random subsampling of the communities at the family level. The dendrogram represents the overall similarity of the sample groups. Yellow colour corresponds to high average Bray-Curtis dissimilarity and black corresponds to the average communities of the sample groups being very similar sample groups. Note that the similarity within subsamples drawn from each group is also indicated in the figure

The total bacterial community compositions in the stool samples showed no systematic significant differences, neither between *Blastocystis* positive and negative individuals, nor between individuals with different subtypes (all *p*-values >0.17 and >0.89, respectively). However, our data for ST3 and ST4 suggest that a relationship to different bacteria still may exist (Fig. 4; Additional file 5: Figure S4). According to the stratification of the human intestinal bacterial flora by enterotypes suggested by Arumugam et al. [21], the PCA in Fig. 4 is shown in relation to *Bacteroides* and *Prevotella,* the genera suggested to drive the human gut enterotypes. Interestingly, the microbiota composition of individuals with different *Blastocystis* subtypes seems not to be equally distributed with regard to the enterotypes. Most of the faecal samples containing *Blastocystis* ST3 clustered more closely to the *Bacteroides* genus in the PCA than did the samples with ST4. Additionally, the bacterial communities of individuals carrying *Blastocystis* ST2 and ST8 differed the most from the *Blastocystis* negative individuals, and ST2 communities stood out as being highly dissimilar to those associated with ST1, ST3 and ST4 (Fig. 5; Additional file 6: Figure S5 and Additional file 7: Figure S6). For most subtypes, however, the variability in bacterial community composition was high also within groups, with none of the differences indicated above being statistically significant at the overall community level (*p* > 0.17).

## Discussion

This metagenomic study of travellers offers new insights with regard to the stability of *Blastocystis* carriership in the intestine, and the relationship between *Blastocystis* and the bacterial microbiota. In a population of 35 university students, the prevalence of *Blastocystis* was high both before and after intercontinental travel. The distribution of *Blastocystis* subtypes, with representation of ST1–4 and a dominance of ST3 and ST4, was unremarkable for a European setting [9, 22]. Apparently, there was little impact of intercontinental travel on the subtype distribution. Among the ten individuals where a reliable subtype designation could be determined from the metagenomic data before and after travel, each carried the same *Blastocystis* subtype on both occasions, implying a persistence of a specific subtype for three weeks to five months. This persistence was seen despite the common occurrence of travellers’ diarrhoea. No evidence of mixed subtype carriage was found, which is in contrast to a recent study from Ireland where mixed STs were found in 22% of *Blastocystis*-carriers [5]. However, most previous studies have found a rather low frequency of mixed STs [9]. Although the occurrence of mixed STs might be underreported in some studies because of inherent methodological limitations [5], we are confident that the method we have used is a sensitive tool to detect the occurrence of more than one ST. Our findings, with a high prevalence of *Blastocystis* and an absence of mixed subtype carriage raise the question if competition between STs may occur, preventing colonization of a second subtype when one is already present.

The presence of travellers’ diarrhoea and possible, non-examined, co-existing bacterial or viral enteropathogens could act as confounding factors in our metagenomic study of the intestinal microflora. However, the participants of the study reported no antibiotic use and we found that the overall composition of the bacterial gut microbiota of the study participants was only moderately affected by travel. The most notable change was an increase in abundance of families within the phylum Proteobacteria in participants returning from Africa [15]. Comparisons of the bacterial composition at the family level showed no significant systematic differences between *Blastocystis*-carriers and non-carriers or between carriers of different *Blastocystis* subtypes. We did, however, find a positive association between *Blastocystis*-carriage and high bacterial genus richness in the gut (Fig. 3). This strengthens the association between *Blastocystis* and high bacterial diversity that has also been reported in a retrospective metagenomic study by Andersen et al. [23], which included both healthy controls and patients with inflammatory bowel disease, and in a metagenomic study by Audebert et al. [24] that investigated the intestinal microbiota in individuals whose faecal samples had previously been sent for diagnostic parasitological exams. Since a gut microbiota with high diversity is generally considered healthy, *Blastocystis* might be seen as an indicator of gastrointestinal health, an opinion also voiced by other researchers [23–25]. Because we lack dietary data and detailed medical history of the participants in our study we cannot substantiate this assumption any further. It is interesting that the association between *Blastocystis* and genus richness was largely driven by ST4 in our study, and we suggest that subtype analysis is performed in further metagenomic studies of *Blastocystis*.

We also found interesting correlations between *Blastocystis* and certain bacterial genera. Firstly, the relative abundance of bacterial members of the genus *Bacteroides* was lower in *Blastocystis*-carriers compared to non-carriers (Fig. 2c). This was especially prominent for those individuals harbouring *Blastocystis* ST4. A negative correlation between *Blastocystis* and *Bacteroides* was also described in the study by Andersen et al. [23] and in another study using qPCR to quantify major bacterial taxa in the intestine of *Blastocystis* carriers and non-carriers [26]. Such a negative correlation was however not reported in the study by Audebert et al. [24], nor in a previous qPCR-based study [27]. There are several possible explanations to the inverse relationship between *Blastocystis* and *Bacteroides*, including competition for nutrients, competition for a specific ecological niche in the gut, discrepancies in the ability to utilize nutrients in the diet and/or a direct negative effect on *Bacteroides* by *Blastocystis* predation of these bacteria. The *Bacteroides*-driven enterotype has previously been linked to diets high in protein and animal fat, typical of a Western diet [28, 29]. However, our study specifically pinpoints *Blastocystis* ST4 to be associated with low *Bacteroides* abundance, and the high prevalence of ST4 found in Europe and Australia [9] seems contradictory to the theory of the *Bacteroides-*enterotype driven by a typical Western diet. Additional explanatory factors have to be considered to better understand the regional patterns of *Blastocystis* subtype distribution and microbiota composition.

We also observed a strong correlation between *Blastocystis* and the genus *Sporolactobacillus*. These anaerobic bacteria are gram positive, spore-forming, and lactic acid-producing rods [30, 31]. The type species, *S. inulinus*, was first isolated from chicken feed in 1963 [30]. Other species have since been isolated from soil or the rhizosphere of plants [32–35]. In recent years, novel species of *Sporolactobacillus* have been cultured from decaying foodstuffs such as spoiled orange juice (*S. putidus*) [36] and spoiled jelly (*S. pectinovorans*) [37], but to our knowledge there are no publications on the role of *Sporolactobacillus* in the gut microbiota. Because of its lactic acid properties *S. inulinus* has been suggested as a possible probiotic [38]. In general, intestinal lactic acid bacteria are favoured by diets high in non-metabolizable sugars which are typically found in vegetables [39].

Another correlation was found between *Blastocystis* and *Candidatus* Carsonella of the Gammaproteobacteria. *Candidatus* Carsonella ruddii, the only described species of this genus, has a tiny genome and seems to lack genes essential for bacterial life on its own [40]. It is a strict endosymbiont of sap-feeding insects of the family *Psyllidae*, jumping plant-lice [41], which in turn are monophagous, i.e. have a strict host plant specificity [42]. The correlation to *Blastocystis* is enigmatic, and could have several explanations. First of all, *Blastocystis* may be associated with some other, yet undescribed, species of the *Candidatus* Carsonella genus. Indeed, endosymbionts of *Blastocystis* have been described [43], and since the rDNA sequence similarity of species within the *Candidatus* Carsonella is, naturally, not known, we cannot exclude that these matches represent other endosymbiotic species. Another possibility is that the bacteria have been ingested through food contaminated with insect cells.

Several findings in this study raise intriguing questions about the role of diet in acquiring and sustaining *Blastocystis* colonization. Both the positive correlation to *Sporolactobacillus* and the negative correlation to *Bacteroides* could indirectly imply a link between *Blastocystis*-carriership and intake of vegetables. The differences in relative abundance of *Bacteroides* and bacterial diversity in carriers of ST3 and ST4 may indicate that these subtypes are favoured by different diets, or different ecological conditions in the gut, which in turn might explain the rarity of mixed subtype carriage. Because the life cycle of *Blastocystis* is still largely unknown [44, 45] and the transmission to humans in high-income countries with high sanitation standards is enigmatic, an interesting topic for future research is the detection of *Blastocystis* subtypes in different foodstuffs.

## Conclusions

In this metagenomic study of *Blastocystis* and the intestinal microbiota in Swedish travellers, we found that persistent carriage of a specific *Blastocystis* subtype was common during intercontinental travel to low resource regions. *Blastocystis* was positively associated with high intestinal bacterial genus richness and the bacterial genus *Sporolactobacillus* while negatively associated with the bacterial genus *Bacteroides*, possibly linking *Blastocystis* to a healthy microbiota and diets high in vegetables.

## Additional files

Additional file 1: Figure S1. Distribution of *Blastocystis* subtypes overall (A), before travel to Africa (B), before travel to the Indian peninsula (C), after travel to Africa (D) and after travel to the Indian peninsula (E). Note that individuals are remarkably stable before and after travel. ND = not detected, ST = *Blastocystis* subtype. (PDF 52 kb)

Additional file 2: Figure S2. Diversity of the gut communities across individual samples. (A) Genus richness, (B) Shannon diversity, (C) Simpson diversity. Samples are colour coded by the subtype present (ST1: green; ST2: blue; ST3: red; ST4: black; ST8: orange; Not detected: white). (PDF 58 kb)

Additional file 3: Table S1. Bacterial genera with significant correlations (overall adjusted *p*-value <0.001) to the abundance of specific *Blastocystis* subtype (ST) SSU-rDNA (PDF 45 kb)

Additional file 4: Figure S3. Rarefaction curves of 50,000 reads corresponding to the bacterial 16S SSU rRNA gene (as detected by Metaxa2) in each sample. Samples are colour coded by the subtype present (ST1: green; ST2: blue; ST3: red; ST4: black; ST8: orange). (PDF 111 kb)

Additional file 5: Figure S4. Principal Coordinates Analysis (PCoA) of the Bray-Curtis dissimilarities between the bacterial genus composition of the samples. Samples are colour coded by the subtype present (ST1: green; ST2: blue; ST3: red; ST4: black; ST8: orange; Not detected: white). Note the similarity of this pattern to the pattern shown in the PCA of Fig. 4. (PDF 40 kb)

Additional file 6: Figure S5. Similarity between the bacterial community compositions of individuals carrying different *Blastocystis* subtypes, or no *Blastocystis* at all (ND). As in Fig. 5, rows indicate the dissimilarity of the average community of each sample group to the average community of the sample groups of the columns, as assessed by repeated random subsampling of the communities at the phylum level. The dendrogram represents the overall similarity of the sample groups. Yellow colour corresponds to high average Bray-Curtis dissimilarity and black corresponds to the average communities of the sample groups being very similar sample groups. Note that the similarity within subsamples drawn from each group is also indicated in the figure. (PDF 66 kb)

Additional file 7: Figure S6. Pairwise Bray-Curtis dissimilarity of the communities at the phylum (A), family (B) and genus (C) levels. Yellow/white corresponds to more dissimilar communities, while red corresponds to near-identical communities. (PDF 798 kb)

## Abbreviations

BLAST: Basic local alignment search tool
ESBL: Extended spectrum beta-lactamase
FDR: False discovery rate
PCA: Principal component analysis
PCoA: Principal coordinates analysis
qPCR: Real-time polymerase chain reaction
SSU-rDNA: Small subunit ribosomal DNA
ST: Subtype
