# Supplementary material for: The relation between Blastocystis and the intestinal microbiota in Swedish travellers
Source: BMC Microbiol. 2017 Dec 11;17:231. doi: 10.1186/s12866-017-1139-7 (PMC5725903; doi:10.1186/s12866-017-1139-7)
Supplement: Supplementary file 1 — Distribution of Blastocystis subtypes overall (A), before travel to Africa (B), before travel to the Indian peninsula (C), after travel to Africa (D) and after travel to the Indian peninsula (E). Note that individuals are remarkably stable before and after travel. ND = not detected, ST = Blastocystis subtype. (PDF 52 kb) [file 12866_2017_1139_MOESM1_ESM.pdf]

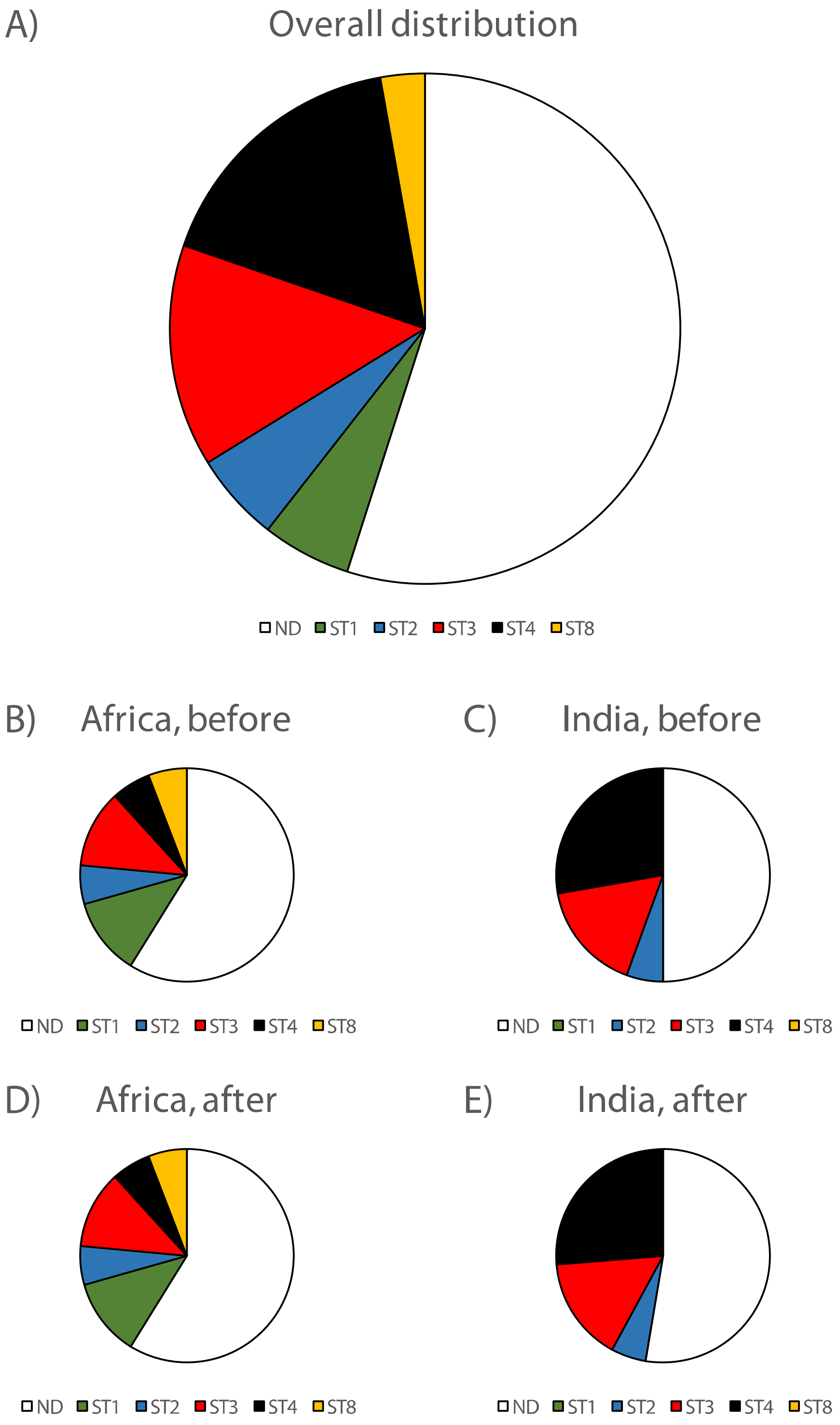

Fig. S1. Distribution of *Blastocystis* subtypes overall (A), before travel to Africa (B), before travel to the Indian peninsula (C), after travel to Africa (D) and after travel to the Indian peninsula (E). Note that individuals are remarkably stable before and after travel. ND = not detected, ST = *Blastocystis* subtype.
