# Supplementary material for: The relation between Blastocystis and the intestinal microbiota in Swedish travellers
Source: BMC Microbiol. 2017 Dec 11;17:231. doi: 10.1186/s12866-017-1139-7 (PMC5725903; doi:10.1186/s12866-017-1139-7)
Supplement: Supplementary file 2 — Diversity of the gut communities across individual samples. (A) Genus richness, (B) Shannon diversity, (C) Simpson diversity. Samples are colour coded by the subtype present (ST1: green; ST2: blue; ST3: red; ST4: black; ST8: orange; Not detected: white). (PDF 58 kb) [file 12866_2017_1139_MOESM2_ESM.pdf]

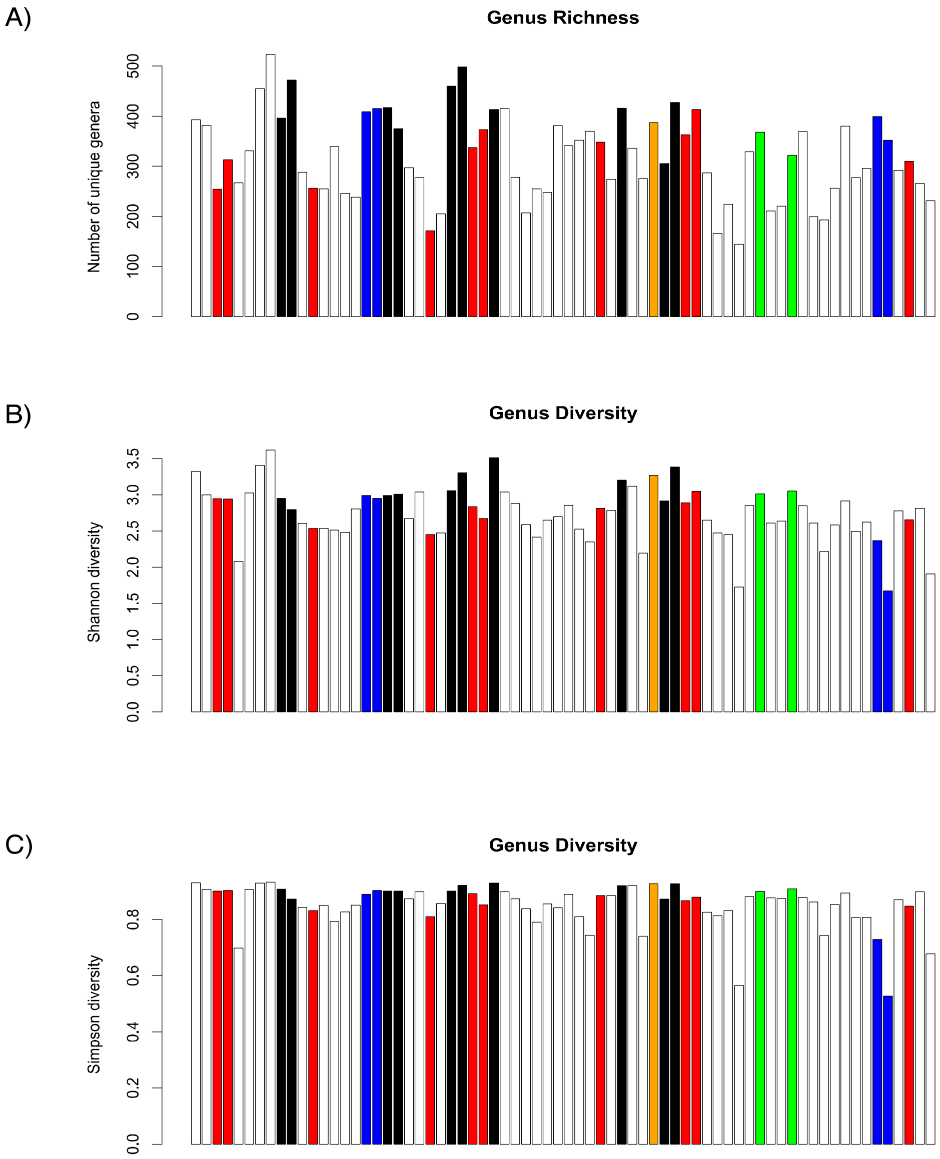

Fig. S2. Diversity of the gut communities across individual samples. (A) Genus richness, (B) Shannon diversity, (C) Simpson diversity. Samples are colour coded by the subtype present (ST1: green; ST2: blue; ST3: red; ST4: black; ST8: orange; Not detected: white).
