# Supplementary material for: The relation between Blastocystis and the intestinal microbiota in Swedish travellers
Source: BMC Microbiol. 2017 Dec 11;17:231. doi: 10.1186/s12866-017-1139-7 (PMC5725903; doi:10.1186/s12866-017-1139-7)
Supplement: Supplementary file 3 — Bacterial genera with significant correlations (overall adjusted p-value <0.001) to the abundance of specific Blastocystis subtype (ST) SSU-rDNA (PDF 45 kb) [file 12866_2017_1139_MOESM3_ESM.pdf]

Supplementary table 1. Bacterial genera with significant correlations (overall adjusted p-value < 0.001) to the abundance of specific *Blastocystis* subtype (ST) SSU-rDNA

Bacteria correlated to *Blastocystis* ST2 (n = 4)	Before travel		After travel		In total	
	Correlation coefficient	Adjusted *p*-value	Correlation coefficient	Adjusted *p*-value	Correlation coefficient	Adjusted *p*-value
Cyanobacteria; Cyanobacteria; SubsectionIII; FamilyI; Lyngbya	0.7174	0.0028	0.7171	0.0020	0.7174	< 0.0001
Proteobacteria; Gammaproteobacteria; Pasteurellales; Pasteurellaceae; Chelonobacter	0.6752	0.0105	0.6752	0.0101	0.6752	< 0.0001

Bacteria correlated to *Blastocystis* ST4 (n = 10)	Before travel		After travel		In total	
	Correlation coefficient	Adjusted *p*-value	Correlation coefficient	Adjusted *p*-value	Correlation coefficient	Adjusted *p*-value
Bacteroidetes; Flavobacteria; Flavobacteriales; Flavobacteriaceae; Polaribacter	0.5575	0.2850	0.7495	0.0008	0.6201	< 0.0001
Proteobacteria; Alphaproteobacteria; Sphingomonadales; Erythrobacteraceae; Unclassified Erythrobacteraceae	0.7197	0.0028	0.5912	0.1486	0.6077	0.0001
Firmicutes; Clostridia; Clostridiales; Family XIV Incertae Sedis; Anaerobranca	0.6516	0.0249	0.5804	0.2043	0.6011	0.0001
Firmicutes; Clostridia; Clostridiales; Family XVI Incertae Sedis; Carboxydocella	0.6279	0.0556	0.5554	0.3364	0.5823	0.0003
Cyanobacteria;Cyanobacteria;SubsectionIV;FamilyI;Anabaena	0.5136	0.6137	0.6854	0.0077	0.5620	0.0009
