# Supplementary material for: The relation between Blastocystis and the intestinal microbiota in Swedish travellers
Source: BMC Microbiol. 2017 Dec 11;17:231. doi: 10.1186/s12866-017-1139-7 (PMC5725903; doi:10.1186/s12866-017-1139-7)
Supplement: Supplementary file 4 — Rarefaction curves of 50,000 reads corresponding to the bacterial 16S SSU rRNA gene (as detected by Metaxa2) in each sample. Samples are colour coded by the subtype present (ST1: green; ST2: blue; ST3: red; ST4: black; ST8: orange). (PDF 111 kb) [file 12866_2017_1139_MOESM4_ESM.pdf]

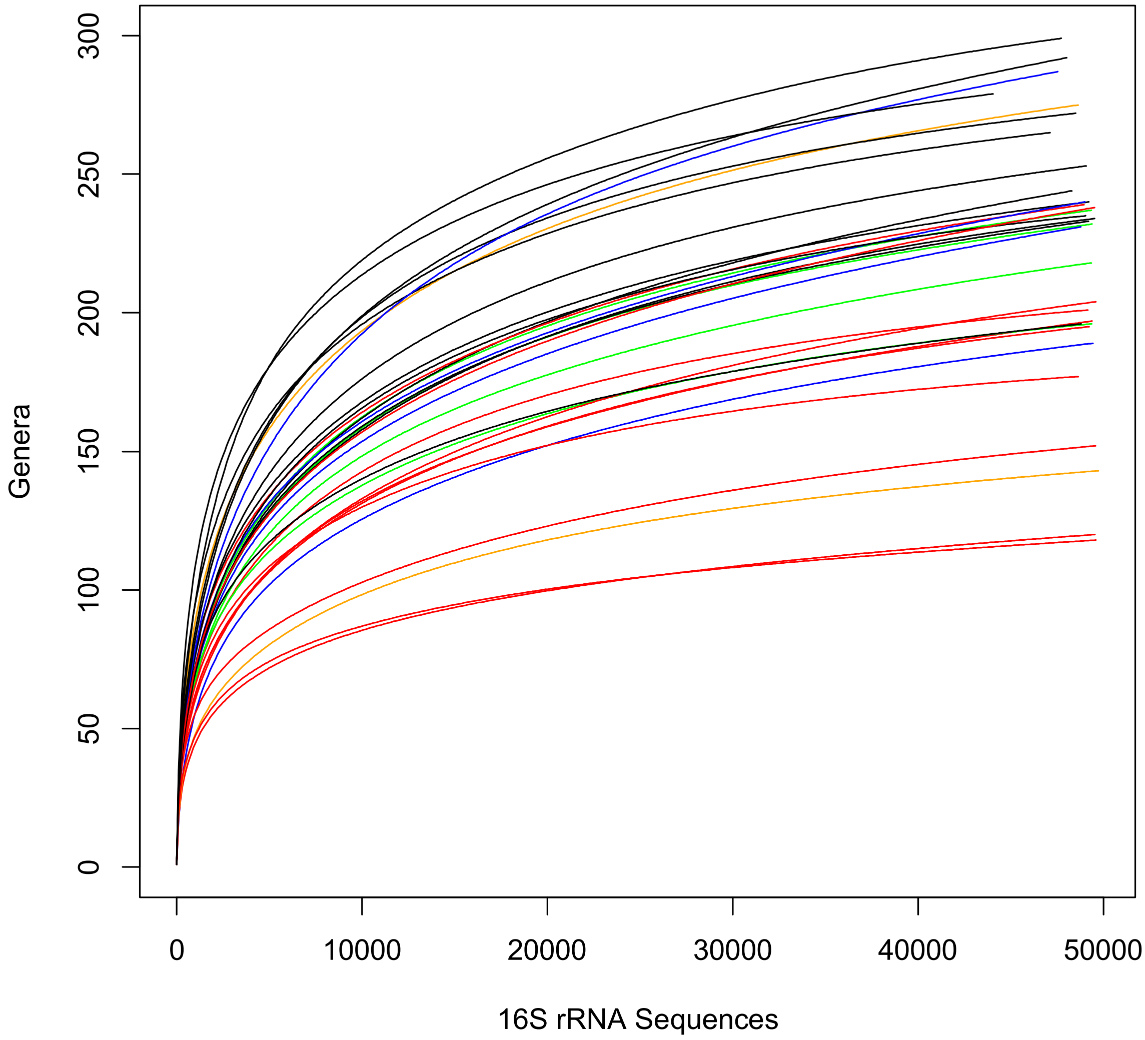

Fig. S3. Rarefaction curves of 50,000 reads corresponding to the bacterial 16S SSU rRNA gene (as detected by Metaxa2) in each sample. Samples are colour coded by the subtype present (ST1: green; ST2: blue; ST3: red; ST4: black; ST8: orange).
