# Supplementary material for: The relation between Blastocystis and the intestinal microbiota in Swedish travellers
Source: BMC Microbiol. 2017 Dec 11;17:231. doi: 10.1186/s12866-017-1139-7 (PMC5725903; doi:10.1186/s12866-017-1139-7)
Supplement: Supplementary file 5 — Principal Coordinates Analysis (PCoA) of the Bray-Curtis dissimilarities between the bacterial genus composition of the samples. Samples are colour coded by the subtype present (ST1: green; ST2: blue; ST3: red; ST4: black; ST8: orange; Not detected: white). Note the similarity of this pattern to the pattern shown in the PCA of Fig. 4. (PDF 40 kb) [file 12866_2017_1139_MOESM5_ESM.pdf]

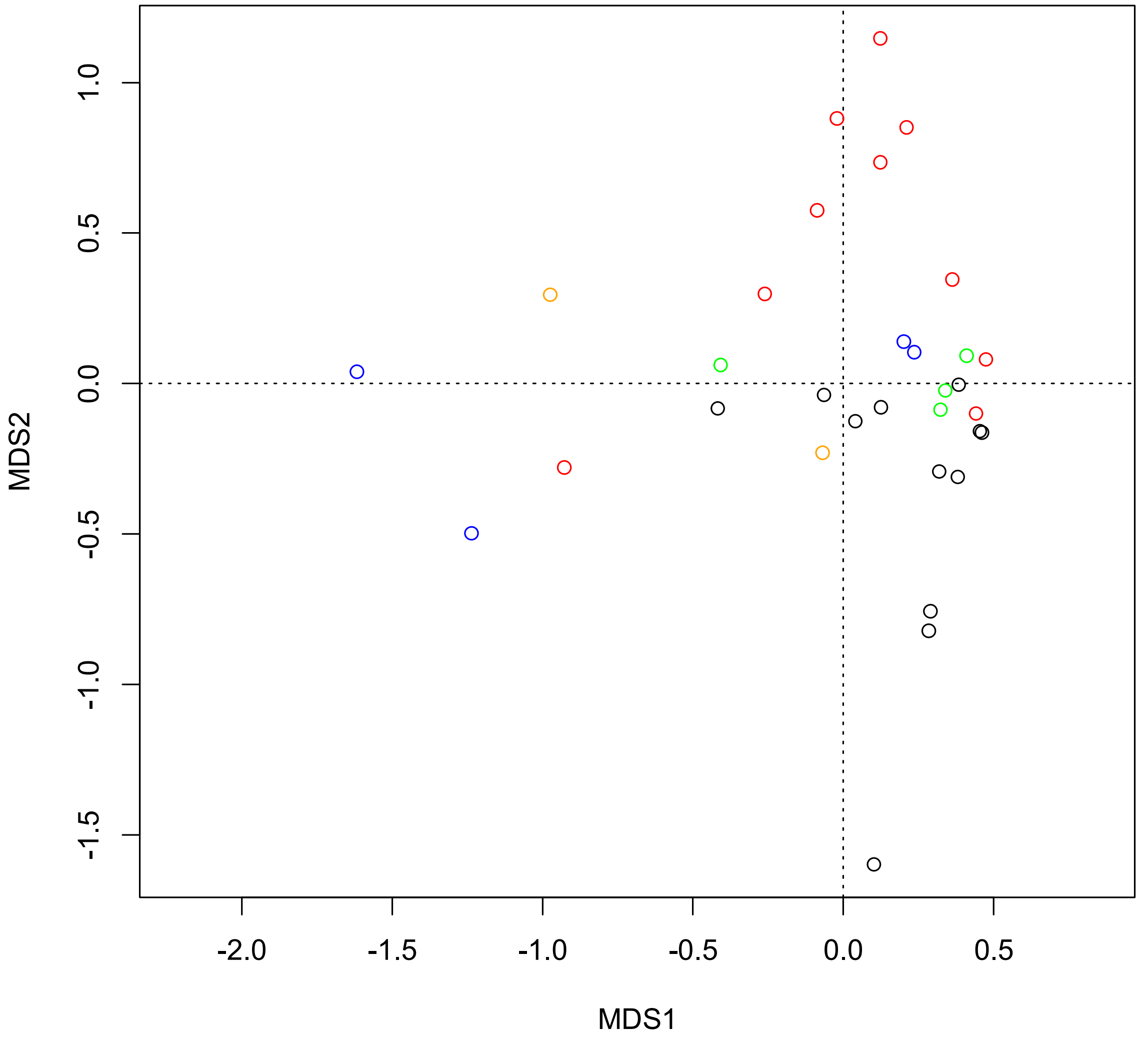

Fig. S4. Principal Coordinates Analysis (PCoA) of the Bray-Curtis dissimilarities between the bacterial genus composition of the samples. Samples are color coded by the subtype present (ST1: green; ST2: blue; ST3: red; ST4: black; ST8: orange; Not detected: white). Note the similarity of this pattern to the pattern shown in the PCA of Figure 4.
