# Supplementary material for: The relation between Blastocystis and the intestinal microbiota in Swedish travellers
Source: BMC Microbiol. 2017 Dec 11;17:231. doi: 10.1186/s12866-017-1139-7 (PMC5725903; doi:10.1186/s12866-017-1139-7)
Supplement: Supplementary file 6 — Similarity between the bacterial community compositions of individuals carrying different Blastocystis subtypes, or no Blastocystis at all (ND). As in Fig. 5, rows indicate the dissimilarity of the average community of each sample group to the average community of the sample groups of the columns, as assessed by repeated random subsampling of the communities at the phylum level. The dendrogram represents the overall similarity of the sample groups. Yellow colour corresponds to high average Bray-Curtis dissimilarity and black corresponds to the average communities of the sample groups being very similar sample groups. Note that the similarity within subsamples drawn from each group is also indicated in the figure. (PDF 66 kb) [file 12866_2017_1139_MOESM6_ESM.pdf]

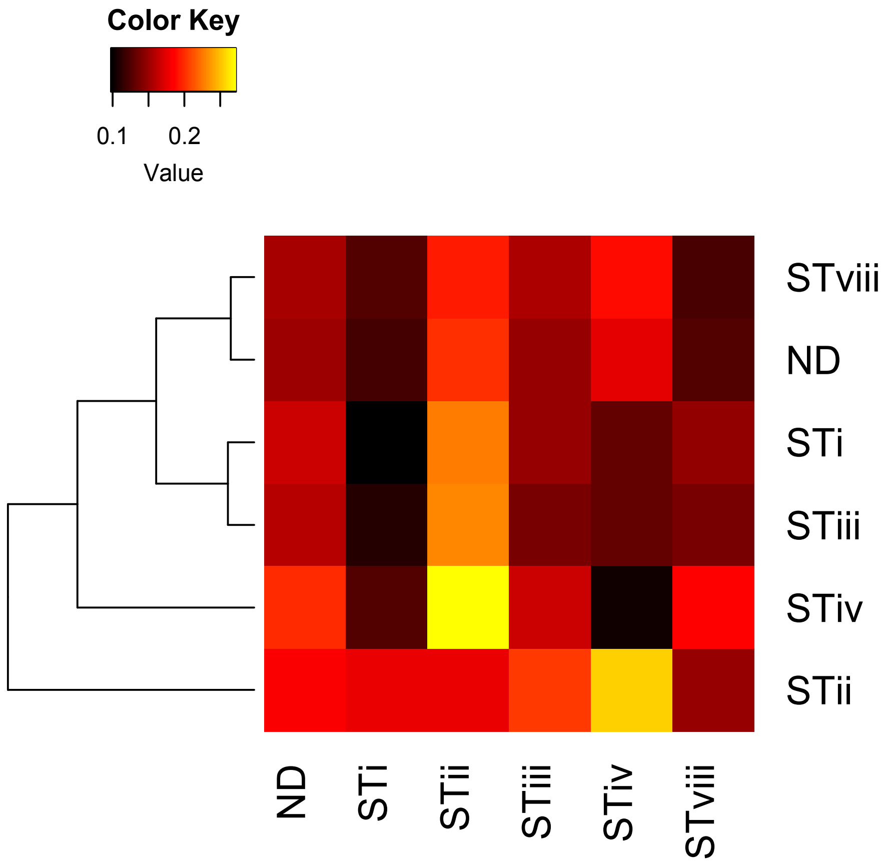

Fig. S5. Similarity between the bacterial community compositions of individuals carrying different *Blastocystis* subtypes, or no *Blastocystis* at all (ND). As in Figure 5, rows indicate the dissimilarity of the average community of each sample group to the average community of the sample groups of the columns, as assessed by repeated random subsampling of the communities at the phylum level. The dendrogram represents the overall similarity of the sample groups. Yellow color corresponds to high average Bray-Curtis dissimilarity and black corresponds to the average communities of the sample groups being very similar sample groups. Note that the similarity within subsamples drawn from each group is also indicated in the figure.
