# Supplementary material for: The relation between Blastocystis and the intestinal microbiota in Swedish travellers
Source: BMC Microbiol. 2017 Dec 11;17:231. doi: 10.1186/s12866-017-1139-7 (PMC5725903; doi:10.1186/s12866-017-1139-7)
Supplement: Supplementary file 7 — Pairwise Bray-Curtis dissimilarity of the communities at the phylum (A), family (B) and genus (C) levels. Yellow/white corresponds to more dissimilar communities, while red corresponds to near-identical communities. (PDF 798 kb) [file 12866_2017_1139_MOESM7_ESM.pdf]

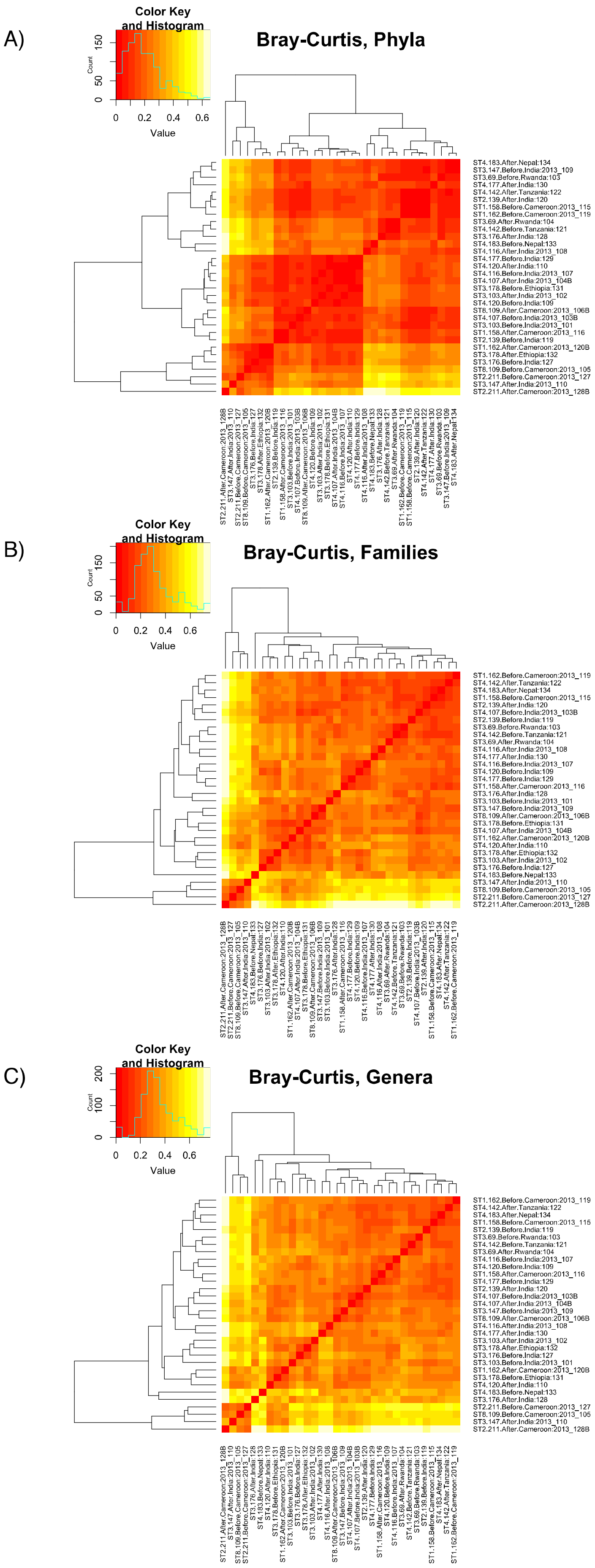
A)
Color Key and Histogram
Count
Value
Bray-Curtis, Phyla
B)
Color Key and Histogram
Count
Value
Bray-Curtis, Families
C)
Color Key and Histogram
Count
Value
Bray-Curtis, Genera

Fig. S6. Pairwise Bray-Curtis dissimilarity of the communities at the phylum (A), family (B) and genus (C) levels. Yellow/white corresponds to more dissimilar communities, while red corresponds to near-identical communities.
